# Supplementary material for: Preservation of collagen in the soft tissues of frozen mammoths
Source: PLoS One. 2021 Oct 29;16(10):e0258699. doi: 10.1371/journal.pone.0258699 (PMC8555803; doi:10.1371/journal.pone.0258699)
Supplement: S1 Raw images — (PDF) [file pone.0258699.s001.pdf]

Raw SDS PAGE gel images

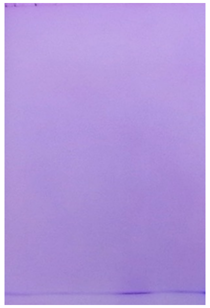

Pepsin fraction
CBB stained

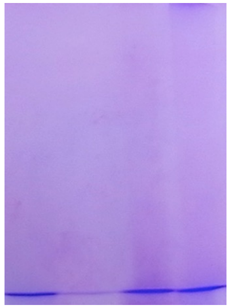

Alkali fraction
CBB stained

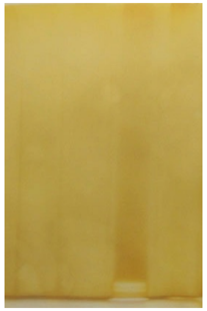

Pepsin fraction
Silver stained

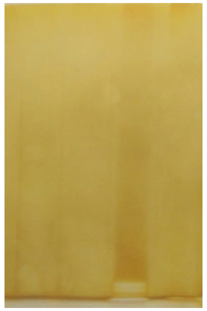

Alkali fraction
Silver stained
