# Supplementary material for: Preservation of collagen in the soft tissues of frozen mammoths
Source: PLoS One. 2021 Oct 29;16(10):e0258699. doi: 10.1371/journal.pone.0258699 (PMC8555803; doi:10.1371/journal.pone.0258699)
Supplement: Attachment — Submitted filename: point by point response.docx [file pone.0258699.s002.docx]

This is resubmission of PONE-D21-11208.

I will describe point-by-point response to the comments provided by the original reviewers

Reviewer #1: This is an excellent paper, very well written and organized. The materials and method section is written comprehensively so that the study can easily be reproduced. The article provides novel information to ECM structure of two frozen baby woolly mammoths that were buried in Siberian permafrost approximately 40,000 years ago.

I appreciate the positive comment for reviewer #1.

Reviewer #2: The authors extracellular matrix (ECM) in the soft tissue of two frozen baby woolly mammoths. It has been indicated that collagenous protein is preserved in the tissues of mammoths (e.g. lung and liver) as a main protein. Generally speaking, the paper would be interesting, however, due to the limited amount of samples, characterization and reported data, it would be suggested to publish this study as a report.

Reviewer #2 pointed out this paper using single measurement for each sample and it is insufficient for statistical analysis. In this submission, I added the new sample of mammoth tissue (genitals)and conducted multiple measurements for each sample. I believe that results become more reliable.

1. I added the Yuki Kumazawa as an author who conducted amino acid analysis.
2. Fig 1 is modified to add the photograph of genitals and elephant tissues as a new sample tissue.

Histochemical observations of mammoth tissues were moved to Fig 2. Because of this change, Numbering of Figs were changed as follows, Fig 2 to 3, Fig 3 to 4, Fig 4 to 5, Fig 5 to 6, Fig 6 to 7 and Fig 7 to 8,

3. In Materials and Methods. I added the genitals tissue from baby mammoth as a amino acid analysis. I used porcine lung tissue instead of bovine lung for amino acid analysis as a fresh tissue control to use tissues from same species origin
4. In Materials and Methods, Amino acid analysis. I conducted multiple measurements for each tissue sample.
5. In Results and discussion, amino acid analysis of tissues.

In table1, I added the data of mammoth genitals. I described the data of porcine lung and purified porcine collagen type I instead of bovine lung and bovine collagen type I. Also added the information source of porcine type I collagen data,

In table 2. I added the data of mammoth genitals. I described the data of porcine lung instead of bovine lung.

In Results and discussion, Fig 6 was changed to average data of multiple measurement and showed standard deviation. I added the data of mammoth genitals. I described the data of porcine lung instead of bovine lung. The description of this result in the manuscript was rewritten using new data.
